# No Evidence for a Saccadic Range Effect for Visually Guided and Memory-Guided Saccades in Simple Saccade-Targeting Tasks

**DOI:** 10.1371/journal.pone.0162449

**Published:** 2016-09-22

**Authors:** Antje Nuthmann, Françoise Vitu, Ralf Engbert, Reinhold Kliegl

**Affiliations:** 1 Psychology Department, School of Philosophy, Psychology and Language Sciences, University of Edinburgh, Edinburgh, United Kingdom; 2 CNRS, Aix-Marseille Université, Marseille, France; 3 Department of Psychology, Division of Experimental and Biological Psychology, University of Potsdam, Potsdam, Germany; 4 Department of Psychology, Division of Cognitive Psychology, University of Potsdam, Potsdam, Germany; University of Leicester, UNITED KINGDOM

## Abstract

Saccades to single targets in peripheral vision are typically characterized by an undershoot bias. Putting this bias to a test, Kapoula [1] used a paradigm in which observers were presented with two different sets of target eccentricities that partially overlapped each other. Her data were suggestive of a saccadic range effect (SRE): There was a tendency for saccades to overshoot close targets and undershoot far targets in a block, suggesting that there was a response bias towards the center of eccentricities in a given block. Our Experiment 1 was a close replication of the original study by Kapoula [1]. In addition, we tested whether the SRE is sensitive to top-down requirements associated with the task, and we also varied the target presentation duration. In Experiments 1 and 2, we expected to replicate the SRE for a visual discrimination task. The simple visual saccade-targeting task in Experiment 3, entailing minimal top-down influence, was expected to elicit a weaker SRE. Voluntary saccades to remembered target locations in Experiment 3 were expected to elicit the strongest SRE. Contrary to these predictions, we did not observe a SRE in any of the tasks. Our findings complement the results reported by Gillen et al. [2] who failed to find the effect in a saccade-targeting task with a very brief target presentation. Together, these results suggest that unlike arm movements, saccadic eye movements are not biased towards making saccades of a constant, optimal amplitude for the task.

## Introduction

The most frequent movements we make in our daily lives are eye movements that bring the fovea to a target of interest (i.e., prosaccades). Since it first became possible to precisely measure the position of the eyes, researchers have been investigating the properties of saccades to single targets in peripheral vision. Thirty years ago, Kapoula [1] reported an intriguing phenomenon: When exposed to a certain range of target eccentricities, oculomotor responses showed a bias towards the center of the range; as a result, saccades overshot targets displayed at the smallest eccentricities in a given block of trials, while they undershot the furthest targets in a block. The replicability of this saccadic range effect (SRE) has been recently called into question [2]. Here, we further challenge the original findings by reporting results from three experiments in which we failed to find reliable evidence for a SRE.

### Weak evidence for a Saccadic Range Effect for Prosaccades, Antisaccades, and Memory-Guided Saccades

The range-error concept was originally proposed by Poulton [3, 4] to account for the influence of the set of conditions in a block of trials on perceptual, motor and memory performances. In manual tracking tasks, for example, small distances were overestimated whereas large distances were underestimated, hence suggesting a response bias towards the center of the range of movement amplitudes in the tasks [5–7]. Assuming that the range effect is a fundamental characteristic of motor skills, Poulton proposed that such a response bias would generalize to saccadic eye movements, but he never tested it directly.

An explicit test of the SRE hypothesis requires an evaluation of saccade accuracy for a given target eccentricity presented within separate blocks of trials covering different (but partially overlapping) ranges of target eccentricities. This was done in very few studies only. Kapoula [1] (see also [8]) reported evidence for a SRE. In her study, saccades tended to overshoot 7° targets when 7° was the smallest eccentricity in the block of trials whereas they were precise in conditions where 6.1° corresponded to the center of the range of possible eccentricities. In contrast, neither Findlay [9] nor Gillen et al. [2] found a SRE. In Findlay’s study, the accuracy of saccades directed at a 3° target did not differ between instances where the 3° target was mixed in a block of trials with stimuli presented at smaller eccentricities (1° and 2°) or greater and smaller eccentricities (5° and 1°), respectively. In the study by Gillen et al. [2], observers completed two blocks with two common eccentricities (10.5° and 13°, which corresponded either to the upper or the lower end of the range of possible eccentricities). The data showed no evidence for a SRE, but rather an undershooting bias across all target eccentricities.

In subsequent work, Kapoula suggested that the SRE is a cognitive strategy to reduce overall variability in saccade accuracy [10]. If that is the case, it should be at work in oculomotor paradigms that are cognitively more demanding than the simple task of making a prosaccade to a single target. In line with this reasoning, the SRE hypothesis has been tested using the antisaccade task, which requires suppressing a stimulus-driven prosaccade. Specifically, the observer is asked to make the saccade towards the target’s mirror location in the opposite hemifield [11]. Two studies have reported a pattern of saccadic dysmetria for antisaccades but not for prosaccades [12, 13]. Both studies used a single block of proximal target eccentricities (Dafoe et al.: 0.5, 1, 2, 4, and 8°; Evdokimidis et al.: 2 to 10° in steps of 1°). Antisaccades in these studies elicited a respective over- and undershooting bias for the near and far targets, whereas there was no bias for the central target. This could be seen as (tentative) evidence that the top-down nature of antisaccades produces a SRE. However, key to the SRE is that saccadic responses to a given target eccentricity depend on the magnitude of eccentricities in a given block. To test this, Gillen and Heath [14] compared a block with proximal eccentricities (range: 3 to 13°) to a block with distal eccentricities (range: 10.5 to 20°). Their results for the proximal block mirrored the findings by Dafoe et al. [13] and Evdokimidis et al. [12]. In the distal block, however, there was a numerical undershoot for the smallest eccentricity and a significant undershooting bias for all other eccentricities. Importantly, 13° targets produced an undershooting bias independent of the block in which they were performed. Consequently, the results from the two blocks together are incompatible with the SRE hypothesis.

Another way to increase the cognitive load associated with the task is to ask participants to move their eyes to a memorized target location. Israel [15] obtained data on memory-guided saccades for six target eccentricities ranging between 5 and 30°, using two different delays (2 vs. 12 s). Her report mentioned that the data showed a SRE for memory-guided saccades, and that no SRE was found when subjects (*N* = 2) acquired information about the targets’ location through visually guided saccades. However, no data and statistics were presented. Critically, her design did not fulfill the criteria outlined above as she only tested a single block of target eccentricities.

### Effects of Target Eccentricity on Saccadic Accuracy for Prosaccades

Compared with the few studies testing the SRE hypothesis, a larger number of studies tested the effect of target eccentricity on saccadic accuracy by using a single block of target eccentricities. A number of studies suggested that the first saccadic eye movement is oftentimes too short to reach the target ([16] for a review). For large eccentricities (> 15°), a systematic undershoot bias of 10% of the distance to the target was reported (e.g., [17]). Others found that the undershooting bias tended to increase with increasing target eccentricity (3–20.5° in [2]; 10–25° in [18]). For smaller eccentricities (3–9°), an undershoot bias of about 5% was found for eccentricities greater than or equal to 6°, whereas mean saccadic responses for shorter eccentricities were accurate [19]. Results from a study in which eccentricities ranging between 5 and 45° were tested showed that only targets beyond 10–15° eccentricity were undershot, and that the amount of undershoot increased with target eccentricity [20]. To account for the undershoot bias, it has been proposed that prosaccades are controlled by an oculomotor control strategy that is designed to minimize saccade flight time [21] or energy expenditure [16].

For close targets at 1°, Findlay [9] observed a high accuracy whereas Kalesnykas and Hallett [22] reported hypermetric (overshoot) responses to a barely visible target at eccentricities between 0.5 and 2°. In a visual discrimination condition, but not in a tracking task, Kapoula and Robinson [23] found a 0.12° overshoot of targets at 5° whereas targets at 10, 15, and 20° were consistently undershot. Their data suggested that undershooting is not an inevitable property of the saccadic system. The authors also interpret their data as further evidence for the SRE. However, following the logic above, this is not the case because finding an eccentricity-specific bias in a single block of target eccentricities is a necessary but not a sufficient condition for the SRE.

### Outline of the Present Study

The findings by Kapoula [1] have been frequently cited (100 citations in Web of Science, 140 in Google Scholar; July 2016), which demonstrates general recognition for a SRE in the literature. Finding evidence for a SRE has important theoretical implications. For one, it would suggest that the control of eye movements is similar to the control of ballistic arm movements, thereby lending support to the view that saccades are also subject to some general muscle-control bias, or else that their programming is influenced by cognitive strategies contingent on the specific set of conditions in a given experimental situation. Moreover, finding a SRE would go against the rather general finding that saccades’ landing position errors systematically take the form of an undershoot; hence, it would argue against the well-accepted hypothesis that saccade amplitude is strategically adjusted so as to minimize the likelihood that the eyes land past the target for economy purposes [16, 21]. Finally, whether or not the phenomenon exists has implications for theories of eye-movement control in reading, an issue we will discuss in depth in the General Discussion.

In recent years, issues relating to the replicability of research findings in psychology have received an increasing amount of attention [24–26]. Factors that contribute to the issues are failures to replicate earlier research even when it is based on stronger data or methodology and the absence of incentives to publish high-quality null results [24, 27].

Our test of the SRE hypothesis consists of three experiments. Experiment 1 is a close replication of the original study by Kapoula [1]. Experiment 2 is a replication study with an extended design. Experiment 3 is a replication-extension study, which was designed to examine conditions that may (or may not) elicit a SRE. In all three experiments we used visually guided saccade tasks. Experiment 3 further included a memory-guided saccade task. In the remainder of this section, we will briefly describe the experiments, summarize and motivate the variables manipulated across experiments, and derive the corresponding predictions.

#### Three experiments

No replications in psychology can be absolutely exact recreations of the original study [28]. Therefore, we refer to Experiment 1 as a close replication of the study by Kapoula [1], and carefully document differences between the replication and the original study. Participants first fixated a central spot. After a variable interval the central spot was turned off and a small square frame containing a variable number of dots was presented. The square appeared in a random manner at variable eccentricities left or right from the central spot. Participants were instructed to move their eyes to the square as soon as it appeared and to report the number of dots in the square. The target was turned off 100 ms after the onset of a saccade was detected. As in Kapoula [1], there were 16 trials per eccentricity condition, and four participants were tested. Kapoula [1] tested four subjects with the first set of eccentricities (*N* = 16 trials per eccentricity condition); two of these subjects also completed the second set of targets (now *N* = 32 trials per eccentricity condition). We kept the number of participants and the number of trials per eccentricity condition constant for the two eccentricity blocks. Kapoula [1] compared two partially overlapping sets of eccentricities [proximal: 2.7, 4.4, 6.1, 7.8 and 9.5°; distal: 7, 10.9, 14.7, 18.3, 21.9°]. We used eccentricities that were 2.5° apart in two blocks with overlapping eccentricities [proximal: 2.5, 5, 7.5, 10 and 12.5°; distal: 7.5, 10, 12.5, 15, and 17.5°]. Thus, there were three common eccentricities in each block (7.5, 10 and 12.5°). In the original study [1], a tangent display oscilloscope was used for stimulus presentation, and a magnetic search coil device [29] for eye-movement recordings. Such equipment was not available to us anymore. Instead, we used a cathode ray tube monitor together with the video-based SR Research EyeLink II eye-tracking system. The search coil is considered the gold standard for eye tracking (but see [30]), although it comes at the cost of being invasive. Several studies have compared the EyeLink I at 250 Hz [31, 32], the EyeLink II at 500 Hz [33] and the EyeLink 1000 [34, 35] with the search coil method [29]. All reports found substantial agreement between the two systems ([35] for review).

Experiment 2 was a replication study with an extended design. First, we added a block of short eccentricities, leading to three sets of eccentricities [short: .5, 1.5, 2.5, 5, and 7.5°; medium (proximal in Exp. 1): 2.5, 5, 7.5, 10 and 12.5°; long (distal in Exp. 1): 7.5, 10, 12.5, 15, and 17.5°]. Second, in comparison to Experiment 1 statistical power was considerably increased in Experiment 2 by testing 10 participants who each contributed a maximum of 50 data points to each block × eccentricity condition. Our replication sample size (*N* = 10) is 2.5 times the original sample size (*N* = 4), which is in agreement with a proposal by Simonsohn [36]. Testing more participants with more trials also allowed for statistical analyses of average performance as well as distributional analyses. Finally, the target was visible for 500 ms (see below).

Experiment 3 combined the three sets of target eccentricities used in Experiment 2 with a manipulation of the cognitive demands imposed by the task. Specifically, the aim was to make the task cognitively more or less demanding than in the previous experiments. For the low-demand condition, the visual discrimination task used in Experiments 1 and 2 was dropped, turning the task into a pure visual saccade-targeting task [2, 23]. A small square frame served again as the target. There were no dots inside the square, and participants were simply asked to move their eyes to the target, which remained visible for 2 s. A memory-guided saccade task [37, 38] served as the high-demand condition. The target was presented for 2 s to allow for sufficient time to encode the target location. This was followed by a 2-s delay (cf. [15, 39]), which introduced visuo-spatial working memory load. The offset of the central fixation dot was the signal for participants to move their eyes to the remembered target location.

#### Summary of manipulations

Starting with a close replication of Kapoula [1] in Experiment 1, a number of variables were manipulated across experiments (Table 1, for visually guided saccade tasks). First, the task given to the participants was varied. In all tasks participants were asked to direct their gaze to the location of an extrafoveally presented target. In Experiments 1 and 2, participants were given a visual discrimination task, i.e., dot enumeration [1]. In addition, in the memory-guided saccade task included in Experiment 3 participants were not allowed to make a saccade before a 2-s delay period was over; they had to memorize the location of the target before making a saccade to the remembered location. Experiment 3 also contrasted the memory-guided condition with a simple visual saccade-targeting task [23] entailing minimal top-down influence. Thus, the saccade aiming tasks used across the experiments varied in the degree of top-down control.

**Table 1:** Comparison of parameters for visually guided saccade tasks across experiments.

Experiment	Task	Number of Eccentricity Ranges	Number of Subjects	Trials per Eccentricity Condition	Target Presentation Duration (ms)
1	Visual Discrimination	2	4	16	Saccade latency + 100
2	Visual Discrimination	3	10	50	500
3	Saccade Targeting	3	12	24	2000

Second, the target presentation time was varied. In Kapoula [1], the target was erased 100 ms after saccade onset detection. Consequently, the target was visible for a variable amount of time, averaging 264 ms, and under the control of participants. This was done “for purposes unrelated to this part of the experiment” (p. 1156). We prefer a fixed presentation time, but for replication purposes this implementation was adopted in Experiment 1. In Experiment 2, the target was presented for 500 ms, i.e., it was visible for longer and for a fixed period of time. Target presentation duration was chosen such that visual feedback from the target itself was available for the programming of both primary and secondary saccades, allowing for testing the SRE hypothesis both through properties of the primary saccade [1] or indirectly through corrective saccades [23]. In Experiment 3, the target presentation time was increased to 2 s; the only motivation for choosing this particular value was to present the target for the same amount of time in both visually guided and memory-guided trials. Compared with Experiments 1 and 2, the visually guided trials in Experiment 3 provided prolonged visual feedback about the target location, whereas no visual feedback was available in the response phase of memory-guided trials.

In comparison with Kapoula [1], Experiments 2 and 3 also incorporated improvements to the experimental design. We tested three sets of eccentricities instead of two. This way, saccade accuracy could be compared between blocks of trials for five rather than two [1] or three (Exp. 1) target eccentricities. Most importantly, targets at 7.5° are a particularly strong test of the SRE hypothesis because we use this eccentricity both in the context of blocks with shorter and larger eccentricities to induce overshoot and undershoot, respectively. Moreover, Experiments 2 and 3 tested 10 (Exp. 2) and 12 (Exp 3) rather than 4 [1] or 5 [23] participants to increase statistical power and to assess the generalizability of findings.

#### Predictions

**Fig 1:**
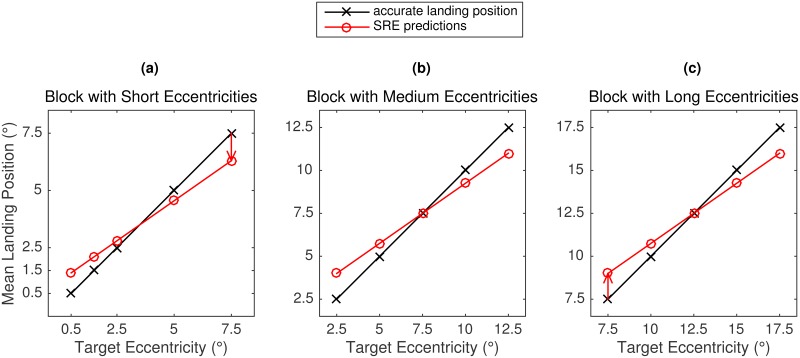
Saccadic range effect hypothesis. Predicted systematic error in the spatial accuracy of saccades to targets at varying eccentricities. Each panel displays predictions for one block of eccentricities. In each panel, the crosses on the black line represent accurate landing positions. The red circles on the red line depict the predicted mean landing position. According to the SRE hypothesis, saccadic responses to a given target eccentricity should depend on the magnitude of eccentricities in a given block. Thus, depending on the context a target displayed at an eccentricity of 7.5° is expected to elicit undershoot, accurate, or overshoot responses. Note that the Figure refers to the three blocks used in Experiments 2 and 3.

According to the SRE hypothesis, if the oculomotor system has a response bias towards the center of a given range of eccentricities, then it should overestimate small target eccentricities and underestimate large target eccentricities for a specific target set. If this is the case, mean landing positions and the distributions of initial landing sites should vary depending on the target eccentricity and block series. In general, targets at close eccentricities in a block should be overshot whereas targets at far eccentricities should be undershot (Fig 1). Furthermore, results for a given target eccentricity should depend on the block series. For example, in Experiment 1 the 10° target eccentricity is common to each block and should elicit a systematic under- and overshooting bias in the proximal and distal blocks, respectively (Fig 1b and 1c). In Experiments 2 and 3, 7.5° targets presented a particularly strong test of the SRE hypothesis. Being in the middle of the eccentricity range in the Medium Block, 7.5° targets should elicit accurate responses (Fig 1b); being the largest eccentricity in the Short Block, 7.5° targets should elicit undershoot responses (Fig 1a); being the closest eccentricity in the Long Block, 7.5° targets should elicit overshoot responses (Fig 1c).

In contrast, if there is no SRE, the distributions associated with each target eccentricity should be very similar across the three blocks of trials. Based on the literature reviewed above, the data may reveal systematic undershoot for the largest eccentricities only, and accurate responses otherwise (e.g., [20]). Alternatively, the data may reveal an overshoot of very close (< 1.5°) and undershoot of very far (> 12°) targets (Kalesnykas & Hallett, 1994). Yet another possibility is that the data show a general undershoot tendency [2]. In any case, saccade accuracy should not depend on the block series.

Additional predictions concerning the various task manipulations can be summarized as follows. In Experiment 1 we should replicate the SRE as reported by Kapoula [1]. The range effect “represents the influence of the past history (e.g., learning and expectations) on performance” ([40], p. 1741). According to Kapoula and Robinson [23], the SRE is established rapidly in only a few trials. In Experiment 2, we exposed participants to a greater number of trials than in Experiment 1, which may further strengthen the SRE. Kapoula and Robinson [23] manipulated the presence or absence of a visual discrimination task, and reported that the effect of target eccentricity on saccadic accuracy was weakened in the absence of a visual discrimination task. Based on these results, the expectation is to observe a weaker SRE or perhaps no SRE in the saccade-targeting task in Experiment 3. On the other end of the spectrum, the memory-guided saccade task is the cognitively most demanding task, and the SRE should be most pronounced in this condition. Given that memory-guided saccades are a lot less accurate than visually guided saccades [37, 38], the hypothesized advantage of the SRE is that it allows one to reduce the overall variability and to optimize accuracy for the central location in a given range of target eccentricities (cf. [10]).

## General Method

### Participants

Participants were undergraduate students from the University of Potsdam, Germany. Four participants took part in Experiment 1. Ten different participants (all female; mean age = 22.2 years, *SD* = 2.3 years) took part in Experiment 2. Another 12 new participants (four female; mean age = 24.5 years, *SD* = 5.0 years) contributed to Experiment 3. Participants contributing to Experiment 1 were each tested in one session. In Experiments 2 and 3, participants were tested in three sessions at three different days. Participants received study credit or were paid 5 € (Experiment 1) or 15 € (Experiments 2 and 3). All participants had normal or corrected-to-normal vision and were naïve with respect to the purpose of the study. The Ethics Committee at the Department of Psychology at the University of Potsdam approved the experiments. Participants gave their written informed consent prior to the experiment, which conformed to the tenets of the Declaration of Helsinki.

### Apparatus

Eye movements were recorded with the video-based SR Research EyeLink II system with a high spatial resolution (noise < 0.01°) at a sampling rate of 500 Hz. Stimuli were displayed on a 22” CRT monitor at a resolution of 1024 × 768 and a refresh rate of 110 Hz. A viewing distance of 60 cm was assured by use of a chin rest. The experimental software controlling stimulus display and response collection was implemented in MATLAB (The Mathworks, Inc.), using the Psychophysics Toolbox extensions [41] as well as the Eyelink Toolbox extensions [42].

### Eye-Tracking Procedure

To align eye and screen coordinate systems, a nine-point calibration was conducted, followed by a validation. The calibration grid covered the entire screen area. During the experimental session, re-calibrations were conducted after every 10th trial. Gaze recording and calibration were binocular. Each trial started with the presentation of a central fixation dot, which alerted the participant to direct their gaze to its location. A fixation check was performed, which was deemed successful if the eyes (mean horizontal positions, averaged across both eyes) continuously stayed within an area of 22 × 22 pixels (0.8° × 0.8°) for 100 ms. If this condition was not met, the fixation check timed out after 750 ms. In this case, a drift correction was performed. Failure of three successive drift corrections triggered a re-calibration. In all experiments, there was an inter-trial interval of 1.5 s, which participants could use for eye blinks.

### Analyses

**Fig 2:**
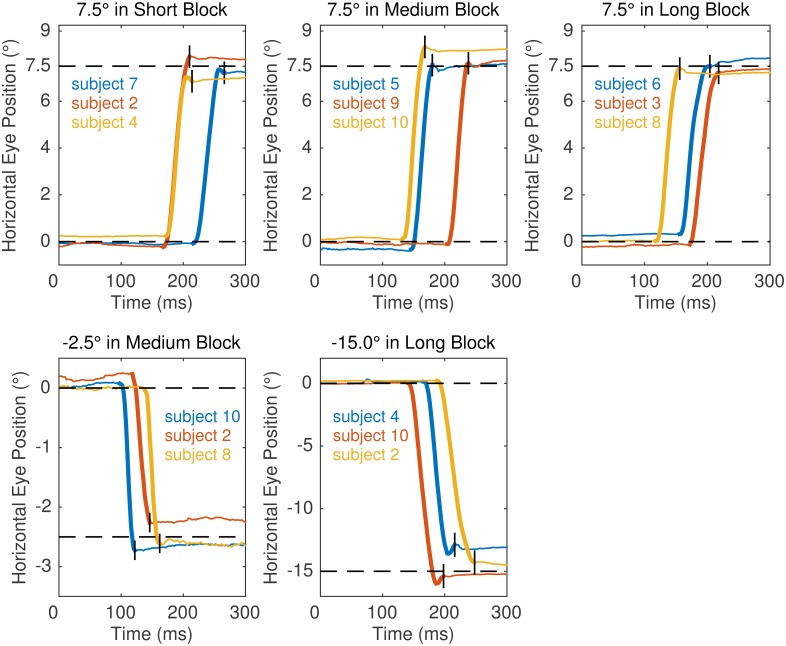
Example gaze traces from Experiment 2. Panels present data for selected target eccentricity conditions from three randomly selected subjects per condition. Horizontal right eye position is plotted for a 300 ms window following the appearance of the target. For each gaze trace, the period identified as a saccade is plotted in bold; in addition, a small vertical black line marks the saccade offset as identified by the algorithm. Veridical target position is indicated by a black horizontal dashed line.

Trials with eye blinks or other errors during data acquisition were discarded. Saccades were detected as rapid binocular eye movements by using a binocular velocity-based detection algorithm that was originally developed for analyses of microsaccades [43, 44], see https://engbertlab.shinyapps.io/Microsaccades. The detection algorithm is sensitive to small eye movements in terms of latency, duration, and amplitude. The time series of eye positions was transformed to velocities with a weighted moving average of velocities over five data samples to suppress noise. Independently for horizontal and vertical components and separately for each trial, a threshold defined as six times the median-based standard deviation of the velocity distribution was computed. When at least four successive velocity samples exceeded the threshold, the sample sequences were classified as saccades. As an additional criterion, the saccade had to occur in both the left and the right eye with a temporal overlap. To demonstrate the quality of the raw data and saccade detection procedure, Fig 2 depicts a number of individual gaze trajectories from the right eye, in which the saccade toward the target is highlighted in bold.

Analyses in Kapoula [1] were based on all saccades, regardless of their latency and the size of the landing-position error. In the present study we made an attempt to distinguish goal-directed saccades towards the target from microsaccades and anticipatory saccades. This was done by taking the amplitude and/or latency of saccades following target presentation into account. For target eccentricities ≥ 2.5°, initial saccades that occurred after target presentation and had a horizontal component smaller than 1° were classified as microsaccades [45]. In this case, the following saccade was treated as initial saccade, unless this saccade was another microsaccade or anticipatory saccade. For the two smallest target eccentricities in Experiments 2 and 3 (0.5°, 1.5°), however, the amplitude criterion was not suitable for distinguishing microsaccades from visually guided saccades; it is possible that saccades with short amplitudes were made in response to target onset. Therefore, in these two conditions the microsaccade criterion was not applied.

On a given trial, the exact time and location of the target’s appearance was not predictable. Even then, participants sometimes make quasi-anticipatory saccades, which can be distinguished from visually guided saccades based on their short latency and direction errors [46]. Therefore, initial saccades were excluded as anticipatory saccades if they moved the eyes in the direction opposite to the target location, or if their latency was shorter than 80 ms.

Data from the right eye were analyzed. If not stated otherwise, the data reported are based on the first (primary) saccade of each trial, regardless of whether subsequent saccades occurred. Data for left and right target presentation were pooled; presentation side had no significant effect. For statistical analyses means were calculated for each subject, and these were then averaged across subjects. Analyses of variance (ANOVA) or *t* tests were run on the means obtained for each subject in each condition. For repeated-measures ANOVAs we report Greenhouse-Geisser corrected degrees of freedom and *p*-values in cases where Mauchly’s test indicated that sphericity was violated.

## Experiment 1

The goal of Experiment 1 was to conduct a close replication of the study by Kapoula [1].

### Method

Each trial started with the presentation of a central fixation dot, which triggered a fixation check (see General Method –Eye-Tracking Procedure, for details). After a stable fixation was achieved, a foreperiod was introduced, whose duration was chosen randomly such that the fixation dot was visible for a variable duration of 750, 950, 1050, or 1250 ms [1]. The fixation dot then disappeared, and the target was presented (with no gap) to either side of the fixation dot along the horizontal axis. Target onset cued participants to move their eyes to the target square box (0.5° on a side) and report the number of internal dots (2 vs. 5) by pressing a key (“n” for two dots, “m” for five dots). As in Kapoula [1], instructions given to the participants stressed speed (“as quickly as possible”) but not accuracy. The target was erased 100 ms after saccade onset detection [1]. To this end, a 5-sample online velocity computation model was implemented, and saccades were identified when gaze data from the right eye reached a velocity threshold of 30 deg/s. Each participant performed two blocks of 80 trials each in one experimental session, which lasted for about 40 min. Depending on the block of trials, the target was presented at different eccentricities:

- Block 1: eccentricities = 2.5, 5, 7.5, 10, and 12.5° (proximal)
- Block 2: eccentricities = 7.5, 10, 12.5, 15, and 17.5° (distal).

Block order was counterbalanced across participants. In the original study, all subjects first completed the proximal block and 2 of the subjects then completed the distal block [1]. We note that the results of a recent study suggest that block ordering does not influence pro- and antisaccade amplitudes [47]. The target appeared equally often in the left and right visual field. Within each block, target eccentricity and visual field (left vs. right) were randomized. Stimuli were presented in black on a white background. Testing took place in a dimly illuminated room.

### Results

Mean accuracy in the discrimination task was 90.25%. Considering a window of 250 ms following online saccade onset detection, trials containing missing data were removed (0.62%). The saccades detected online were validated by comparing them with saccades detected offline. Trials were excluded from analysis if the latency of the online detected saccade was considerably shorter (30 ms or more) than the latency of the offline detected saccade (11.4%). One additional trial was excluded because the first saccade was classified as an anticipatory saccade.

**Fig 3:**
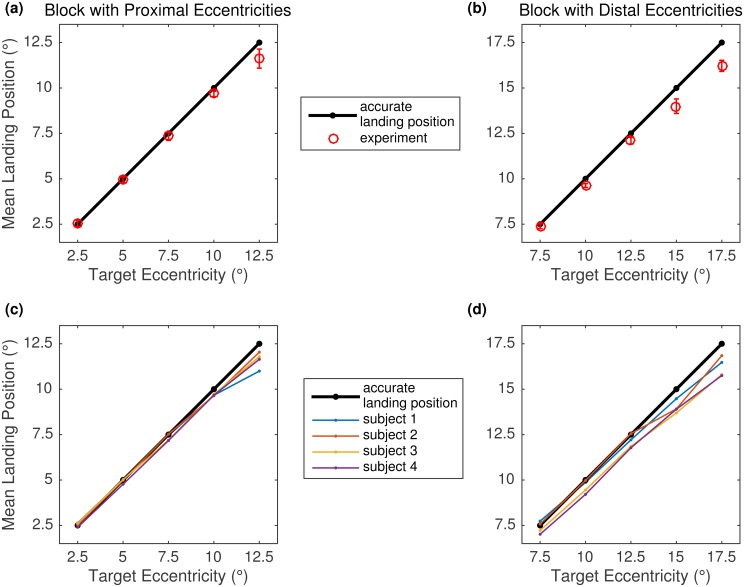
Mean landing positions in Experiment 1. Data are presented as a function of target eccentricity in the proximal (left panels a and c) and distal (right panels b and d) blocks. In each panel, the solid black line and dots represent accurate landing positions. The red circles in the upper panels represent mean landing positions averaged across four participants. Error bars are within-subject 95% confidence intervals, using the Cousineau-Morey method [48, 49]. Complementary, the colored dots and lines in the lower panels provide participant-specific mean landing positions.

To test the SRE hypothesis, mean landing position was computed for each eccentricity in the proximal (Fig 3, left panels) and distal (Fig 3, right panels) blocks. In each panel, the solid black line with dots represents accurate landing positions. The upper panels in Fig 3 provide mean landing positions for target eccentricities in the proximal (panel a) and distal (panel b) blocks, averaged across the four participants. The lower panels in Fig 3 provide participant-specific mean landing positions for each eccentricity in the proximal (panel c) and distal (panel d) blocks.

The results did not support the SRE hypothesis. In a given block, there was no overshoot for small eccentricities. Across the two blocks, mean saccadic endpoints (landing positions) appeared to be precise for eccentricities smaller or equal to 7.5°, whereas larger eccentricities were associated with a numerical undershoot bias. For the 10° and 12.5° target eccentricities, common to each block, mean landing position was indicative of a small numerical undershoot in both the proximal and distal blocks. Since only four participants were tested, the statistical significance of effects was not determined. Low statistical power does not only reduce the chance of detecting a true effect, but it also reduces the likelihood that a statistically significant result reflects a true effect [50].

In Kapoula’s [1] study, mean latency of primary saccades was 168 ms in the proximal block and 159 ms in the distal block. In the present experiment, the corresponding mean saccade latencies were 148 ms and 165 ms, respectively. This suggests that the absence of a SRE in our study was not due to differences in saccade latencies between our study and the study by Kapoula [1].

## Experiment 2

### Method

Experiment 2 added a set of short eccentricities, leading to three sets of eccentricities, which were arranged in three separate blocks. Each block comprised 250 trials, i.e., 50 trials for each of the five eccentricity conditions in a given block. Participants (*N* = 10) were tested in three sessions, each lasting for about an hour. Blocks were completed in the following order:

- Block 1: eccentricities = 2.5, 5, 7.5, 10, and 12.5° (medium)
- Block 2: eccentricities = .5, 1.5, 2.5, 5, and 7.5° (short)
- Block 3: eccentricities = 7.5, 10, 12.5, 15, and 17.5° (long).

The procedure in Experiment 2 was identical to the one used in Experiment 1 except for the target presentation duration. The target was presented for 500 ms, i.e., in comparison to Experiment 1 the target was visible for longer and for a fixed period of time.

### Results

Mean accuracy in the discrimination task was 89%. Throughout the experiment, 3.3% of all trials had to be discarded due to missing data [block 1: 3.1%, block 2: 3.2%, block 3: 3.7%]. For target eccentricities ≥ 2.5°, on average 0.06% of the data were excluded as anticipatory saccades. For eccentricities 0.5° and 1.5° in the Short Block, 3.3% and 3.7% of initial saccades were affected, respectively. This increase is due to the fact that many of the excluded saccades were microsaccades, i.e., these saccades had both a short latency as well as a small amplitude.

Saccadic error in spatial accuracy can be divided into systematic and variable errors [38, 51]. Systematic error is a bias in landing position that is quantified as the mean deviation between the landing position and the veridical target position. A variable error component produces the spread in landing position distributions. In Experiment 2, we tested more participants with more trials, which enabled us to perform statistical analyses on the systematic error component and to analyze the variable error component. In addition, we tested the SRE hypothesis by analyzing corrective saccades.

#### Mean landing positions and distributions of initial landing sites

**Fig 4:**
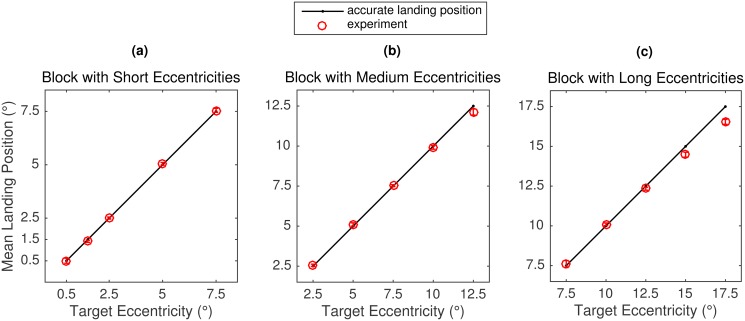
Mean landing positions in Experiment 2. Data (in red) are presented as a function of block and target eccentricity within block. Error bars represent within-subject 95% confidence intervals [48, 49].

To quantify the systematic error, mean landing position was computed as a function of target eccentricity, but separately for the three blocks (Fig 4). Circles represent empirical mean landing positions and vertical bars their standard errors. Results did not support the SRE hypothesis. As shown in Fig 4, there was no overshoot for small distances, not even in the Short Block condition with eccentricities as small as 0.5 and/or 1.5° (Fig 4a). Quite to the contrary, mean saccadic responses were remarkably precise. Only the largest eccentricities in the Medium (12.5°) and Long Block (15°, 17.5°) revealed an undershoot tendency (Fig 4b and 4c). For statistical tests, landing positions were transformed to deviations from target position (0°) coding saccadic undershoot in negative values. Simple *t* tests confirmed significant undershoot responses for three eccentricity conditions: Medium Block: eccentricity 12.5° [mean deviation -0.37° (-3.0% of the distance of the target); *t*(9) = -3.3, *p* = .009], Long Block: eccentricity 15° [-0.53° (-3.5%); *t*(9) = -3.6, *p* = .006] and 17.5° [-0.96° (-5.5%); *t*(9) = -6.6, *p* < .001].

**Fig 5:**
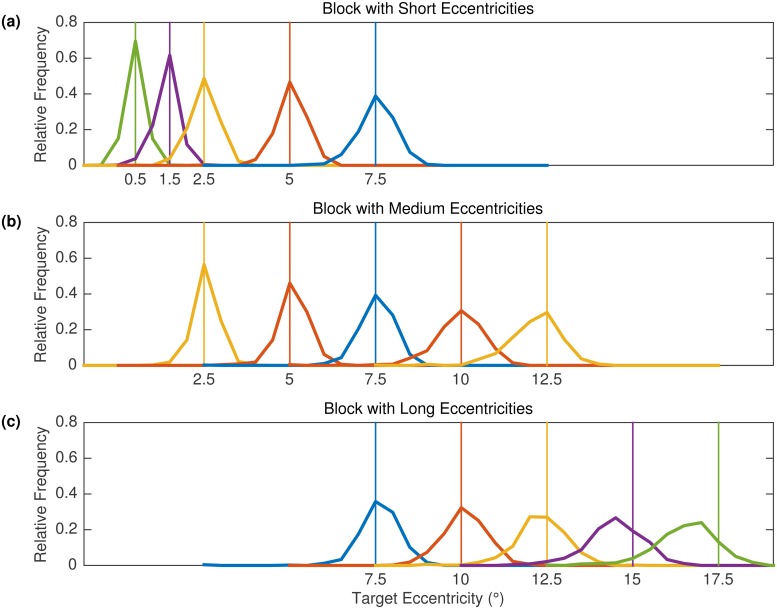
Landing position distributions in Experiment 2. Each panel displays results for one eccentricity block. Data for the same target eccentricities in different blocks are depicted by the same color. Vertical lines represent the veridical target position. Veridical position was defined as the center of the target box, which was 0.5° in width.

To quantify the variable error, Fig 5 shows the distributions of initial landing sites in the different conditions. Landing positions were grouped into 21 bins (width 0.5°) that were symmetrically centered around target position. For most eccentricities, landing positions were narrowly distributed around the veridical target position, with the spread of the distributions increasing with eccentricity. The proportion of target overshoot was no greater than the proportion of undershoots, not even for the smallest target eccentricities. However, there was an undershoot tendency for the 15° and 17.5° eccentricity conditions. In contrast to the SRE hypothesis, the distributions for the 2.5°, 5°, and 7.5° conditions were similar between Short and Medium Block conditions, and there was no apparent difference between Medium and Long Block conditions for the 7.5° and 10° eccentricity conditions, respectively. The 12.5° eccentricity condition was the largest eccentricity in the Medium Block and in the middle of the range in the Long Block. In the Medium Block, the peak of the landing position distribution coincided with the veridical target location. In contrast, the peak of the distribution was slightly shifted in the direction of undershoot in the Long Block. Still, the distribution was more skewed towards the fovea in the Medium Block than in the Long Block, and this is why mean landing positions showed a significant undershoot for the 12.5° eccentricity condition in the Medium Block but not in the Long Block. We note that this undershoot for 12.5° targets in the Medium Block was not replicated in Experiment 3.

To test whether the spread of the landing position distributions significantly increased with target eccentricity, we evaluated participant-specific mean standard deviations of landing positions. Data from each block were analyzed separately by means of a one-way repeated measures ANOVA with target eccentricity as within-subject factor. The eccentricity effect was significant for both the Short Block [*F*(2.13, 19.21) = 22.26, *p* < .001] and the Medium Block [*F*(2.09, 18.78) = 6.01, *p* = .01]. For the Long Block, the effect failed to be significant after Greenhouse-Geisser correction was applied [*F*(1.36, 12.26) = 2.74, *p* = .12].

In the original study, saccade amplitudes were analyzed as the dependent measure [1]. However, if the primary saccade to the target does not have its onset exactly on the starting position at the center of the screen, the amplitude of the saccade is no longer a direct measure of its accuracy (cf. [52]). Specifically, participants’ fixational eye movements (microsaccades, drift, see [53]) may compromise the validity of the saccade-amplitude measure. This problem does not apply here, as we analyzed landing positions directly. Still, control analyses reported in S1 Appendix investigated this issue further and suggested that saccade amplitude is just as good a measure of saccade accuracy as landing position.

#### Saccade latencies

In comparison with Kapoula’s [1] data, our data showed very little systematic error in the spatial accuracy of saccades. It is known that saccadic accuracy increases as saccadic latency increases (e.g., [54–56]). The mean latency for primary saccades, averaged across all blocks, eccentricity conditions and participants, was 153 ms (*SD* = 26 ms), which is similar to the latencies reported by Kapoula [1], see above. Therefore, differences in saccade latencies cannot explain the differences in saccadic accuracy across studies.

**Fig 6:**
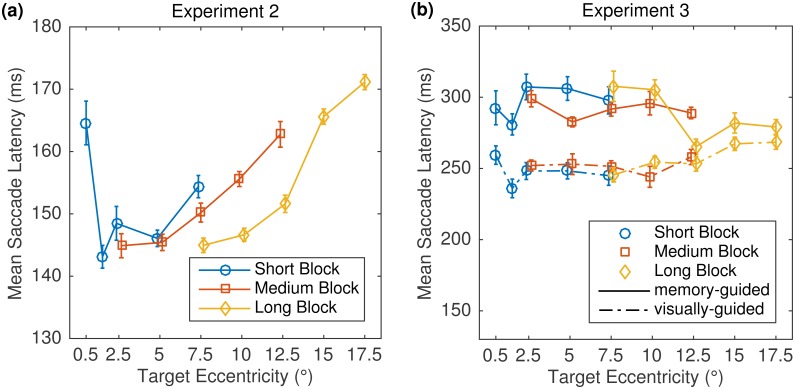
Mean saccade latencies for Experiment 2 (a) and Experiment 3 (b). Data from the three eccentricity blocks are depicted in different colors. Note the different *y*-axis scales for the data from the two experiments. Error bars represent within-subject standard errors [48, 49].

Fig 6a shows mean saccade latency as a function of block and eccentricity condition within block. The data pattern replicates the previously reported bowl-shaped latency-eccentricity function with a foveal latency peak, a minimum plateau from 0.75° to 12°, and a gradual increase in latency towards the periphery [22, 57]. Specifically, we observed considerably longer latencies for the smallest target eccentricity (0.5°) in the Short Block condition, in agreement with previous work [22, 58]. Furthermore, we observed prolonged latencies for the two farthest target positions (15°, and 17.5°) in the Long Block condition, confirming results by Kalesnykas and Hallett [22]. Finally, as shown in Fig 6a, the latencies were longer for eccentricities 10°, and 12.5° in the Medium Block as compared with the Long Block condition. This finding might be due to a practice effect. All subjects started with the Medium Block condition; i.e., block order was not counterbalanced.

#### Characteristics of secondary saccades

In a final set of analyses, the probability and characteristics of secondary saccades were investigated. Secondary saccades are often corrective saccades, i.e., they correct the initial aiming error [59, 60]. Corrective saccades are small-amplitude saccades that quickly follow a primary saccade. They can occur either on the basis of a visual error sampled after the end of the first saccade or on the basis of extra-retinal information [61]. When investigating the influence of target eccentricity on saccadic accuracy, Kapoula and Robinson (1986) conducted an additional indirect test with corrective saccades. They measured the proportion of secondary saccades that moved the eyes in the same direction or in a direction opposite to the primary saccade; these were taken as indicators of under- and overshoot main errors. The authors found that the proportion of overshoot corrections decreased with eccentricity, whereas the proportion of undershoot corrections showed the reverse pattern.

**Fig 7:**
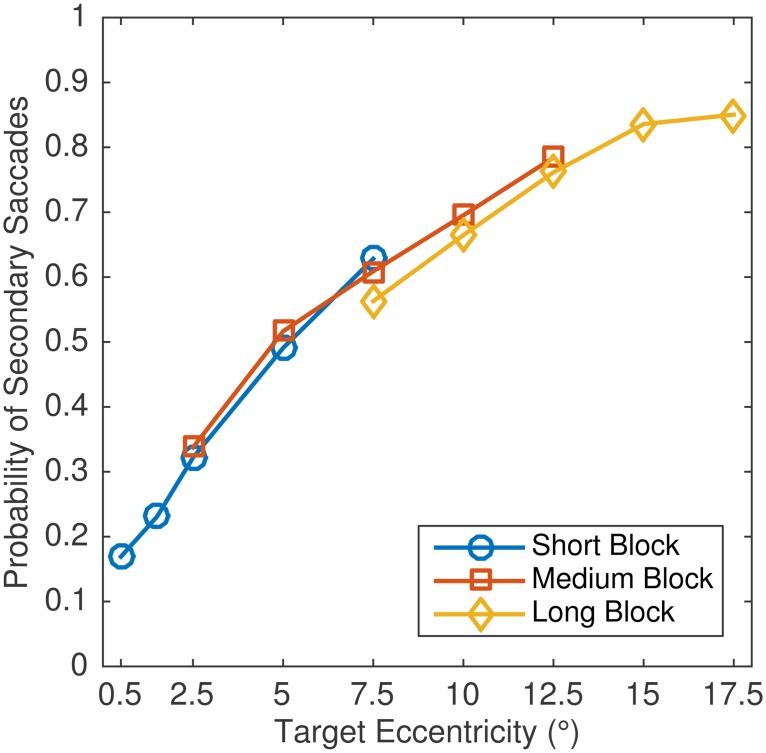
Probability of secondary saccades in Experiment 2. Data are presented as a function of block and eccentricity condition within block.

**Fig 8:**
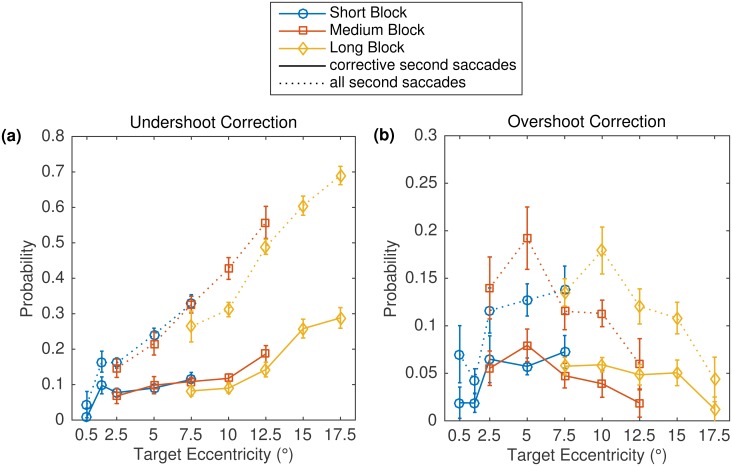
Undershoot Corrections and Overshoot Corrections in Experiment 2. Probabilities of undershoot corrections (a) and overshoot corrections (b) as a function of block and eccentricity condition within block. See text for details. Note the different *y*-axis scales in the two panels. Error bars represent within-subject standard errors.

In the present study, 56% of all visually guided saccades were followed by one or more secondary saccades. The probability of secondary saccades systematically increased with target eccentricity (Fig 7). We then calculated the probability of secondary saccades that were made in the same direction as the primary saccade (undershoot correction) or in a direction opposite to the primary saccade (overshoot correction) for the different conditions. The results revealed no trace of a range effect (Fig 8, dotted lines, representing results for all second saccades). The proportions of undershoot corrections increased with eccentricity in the three block series. Critically, overshoot corrections were most likely for intermediate eccentricities across the three blocks.

**Fig 9:**
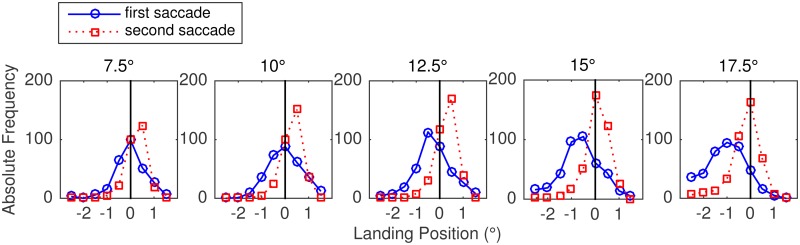
Landing position distributions for first and secondary saccades in the Long Block of Experiment 2. The results for the different eccentricity conditions are presented in different panels. For this analysis, only cases in which more than one saccade was made during the response phase were considered. By comparison, the main analyses were based on all initial (first) saccades, irrespective of whether it was followed by a second saccade or not.

Notably, landing position distributions for first and second saccades showed that not all second saccades were in fact corrective. Exemplarily, Fig 9 shows data from the Long Block. Only in the two conditions in which the primary saccade considerably undershot the target (15°, 17.5°), the second saccade truly corrected the undershoot generated by the primary saccade. We suggest that this was necessitated by the discrimination task, which required landing positions close to the target box. In contrast, if the peak of the distribution for first saccades corresponded to the target position (7.5°, 10°) or a position slightly to the left of it (12.5°), the second saccade showed the tendency to overshoot the target position. It appears that, in these conditions, a lot of second saccades were made for reasons other than correcting the landing-position error of the first saccade.

For further analyses, multiple-fixation cases were classified as undershoot and/or overshoot corrections if the first saccade had placed the eyes outside of the target box (undershoot: in front of the target box; overshoot: beyond the target box) and the second saccade moved the eyes into the target box. As can be seen in Fig 8 (solid lines, representing results for corrective second saccades), the probability of an undershoot correction again increased with target eccentricity, in all three blocks. However, this time the increase was not linear. Rather, there was a plateau up to about the 10° eccentricity condition, and the proportion of undershoot corrections was only increased for the three largest eccentricities (12.5 to 17.5°). Furthermore, the probability of overshoot corrections was not increased for the closest targets in a block. Rather, there were only a few overshoot corrections, and their probability of occurrence remained relatively independent of target eccentricity in all three blocks. This finding is incompatible with the SRE hypothesis.

## Experiment 3

The aim of Experiment 3 was to investigate the SRE as a function of the cognitive load associated with the task. Compared with the previous experiments, we made the saccade-targeting task cognitively more demanding or less demanding. The manipulation of task demands (high vs. low) was crossed with the three sets of target eccentricities used in Experiment 2. In both tasks, the target was presented for 2 s. In the visually guided saccade task (low demand), participants were asked to move their eyes to the target once it appeared. In this task, visual information about the target was present before, during, and (long) after the primary saccade to the target. This was not the case in the memory-guided saccade task (high demand), in which participants were asked to saccade to the memorized target location only after a 2-s delay period, thereby introducing visuo-spatial working memory load.

### Method

The three sets of eccentricities were again arranged in three separate blocks. Each block comprised 120 trials, i.e., 24 trials for each of the five eccentricity conditions in a given block. Participants were tested in three sessions, each lasting for about an hour. Each session tested one range of eccentricities (short vs. medium vs. long). Within each session, participants completed one block of memory-guided saccades and one block of visually guided saccades. The order of sessions and the order of blocks within each session were counterbalanced across the 12 participants. As in Experiments 1 and 2, a small square frame (0.5° × 0.5°) served as the target. It did not contain any dots, due to the specific task demands in Experiment 3. Any lights in the experimental suite were extinguished during the experiment. Stimuli were presented in gray on a black background.

#### Memory-guided saccade task

Each trial started with the presentation of a central dot from the EyeLink calibration routine. It was visible for a variable duration [1]. After a successful fixation check and upon completion of the waiting period, two events happened simultaneously: the target was presented to either side of the fixation stimulus, and a smaller fixation dot (3 × 3 pixels in size or 0.11°) replaced the fixation check stimulus at the center of the screen. Once the target appeared subjects were given 2 s to encode its location in extrafoveal or peripheral vision while maintaining fixation. When the target disappeared, the fixation dot remained on the screen signaling the participant to maintain fixation. After a 2-s delay period, the fixation dot was removed signaling the participant to move his or her eyes to the remembered target location. After another 2 s a beep signaled the end of the trial. The instructions given to the participants were to memorize the location of the target while maintaining fixation until the fixation dot disappeared, and then to make a saccade as fast and accurate as possible to the location where the target had been presented. Participants were given ten practice trials to familiarize them with the task and procedure.

#### Visually guided saccade task

As with the memory-guided saccade task, each trial started with the presentation of a central fixation dot, which was visible for a variable duration. The fixation dot then disappeared, and the target was presented (with no gap) to either side of the fixation dot on the horizontal axis. The visual discrimination task used in Experiments 1 and 2 was replaced with a saccade-targeting task [2, 23]. Participants were instructed to move their eyes as quickly and as accurately as possible to the target location [2]. The target remained visible for 2 s. Half a second later a beep signaled the end of the trial (for consistency with the memory-guided saccade trials). Participants were given five practice trials.

### Results

The percentage of trials with missing data averaged 8.73% for memory-guided saccades and 2.20% for visually guided saccades. The memory-guided saccade task required prolonged visual fixation, during which microsaccades are likely to occur [45, 53]. At the same time, trials in which participants made pre-mature goal-directed saccades during target presentation and the subsequent delay period need to be excluded from analysis. Therefore, we accepted trials in which observers made eye movements with a horizontal component smaller than 1° in the critical time window, but rejected trials otherwise. By this criterion, another 9.6% of the trials from the memory-guided saccade task were excluded from analysis. For memory-guided saccades, the offset of the fixation dot indicated the start of the response period. Given that 4 s had passed since target onset, the 80-ms latency-criterion for anticipatory saccades (Section General Method –Analyses) was not applied to the response period. For consistency, and given the very low number of anticipatory saccades in Experiments 1 and 2, we also omitted this criterion for visually guided saccades. However, as in the previous experiments we applied the microsaccade criterion when selecting the primary saccade towards the target during the response phase (Section General Method –Analyses, for details).

**Fig 10:**
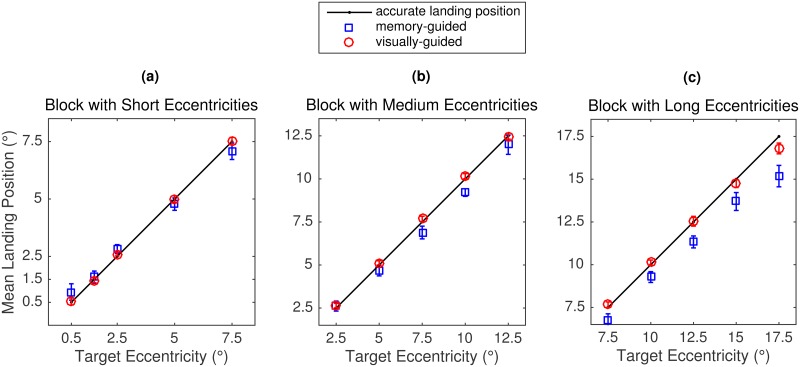
Mean landing positions in Experiment 3. Data are presented as a function of task (red circles: visually guided saccades; blue squares: memory-guided saccades), block (left to right panels), and target eccentricity within block. Error bars represent within-subject 95% confidence intervals.

**Fig 11:**
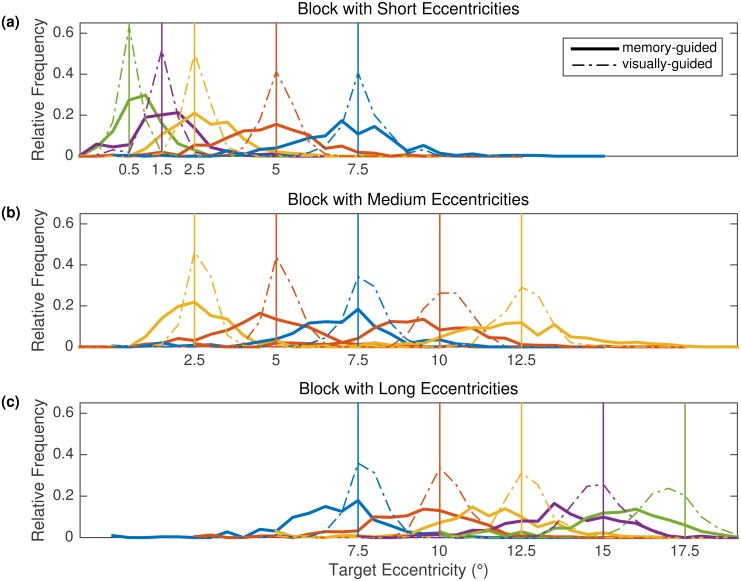
Landing position distributions in Experiment 3. Data are presented as a function of task, block, and target eccentricity condition within block. Each panel displays results for one block. Bold solid lines represent memory-guided saccades, dash-dotted lines visually guided saccades. Vertical lines represent the veridical target positions. Veridical position was defined as the center of the target box, which was 0.5° in width. Data for same eccentricities in different blocks are depicted by the same color.

Analyses focused on the mean landing position of the first saccade towards the target (Fig 10) and the corresponding landing position distributions (Fig 11).

#### Memory-guided saccade task

The most prominent feature of the memory-guided saccades was the enormous spread of landing positions around the veridical target position (Fig 11). The spread of the distribution increased with target eccentricity. Mean landing positions were still close to the veridical target position in the Short and Medium Blocks (Fig 10). The exceptions were as follows: significant overshoot for 0.5° targets in the Short Block [0.450°; *t*(11) = 4.9, *p* < .001] and undershoots for 7.5° [-0.614°; *t*(11) = -2.4, *p* = .034] and 10° [-0.792°; *t*(11) = -3.2, *p* = .008] targets in the Medium Block. Moreover, mean landing positions showed a consistent undershoot tendency in the Long Block (*p* ≤ .029). For the 7.5° targets—common to all three blocks—the mean landing position did not deviate from the veridical target position in the Short Block (SRE prediction: undershoot) and showed an undershoot bias both in the Medium Block (SRE prediction: accurate) and in the Long Block (SRE prediction: overshoot). Taken together, the data pattern obtained for memory-guided saccades is not compatible with the SRE hypothesis.

#### Visually guided saccade task

For 12 out of 15 eccentricity conditions, mean landing position did not differ significantly from the veridical target position (*p* > .05, Fig 10). As in Experiment 2, there were significant undershoot responses for the two largest eccentricities in the Long Block (15°: -0.255° = -1.7%, *t*(11) = -2.4, *p* = .033; 17.5°: -0.700° = -4.0%, *t*(11) = -5.7, *p* < .001). When 2.5° was the closest eccentricity (Medium Block), the mean landing position was indicative of a small numerical overshoot, which was significant at the 5 percent level [0.142°; *t*(11) = 2.5, *p* = .029]. It should be noted that this difference between mean landing position and the center of the target box was not significant if Bonferroni correction for multiple comparisons was applied, as in [2]. The landing position distribution for this condition peaked at the veridical target position, but the distribution was a bit skewed to the right, representing the increased number of overshoots (Fig 11b). By comparison, when the 2.5° condition was in the middle of the range (Short Block), the distribution was symmetrical around the veridical target position (Fig 11a). Together, the data for the 2.5° eccentricity condition showed a hint of a range effect. We note that the data from Experiment 2 did not show this particular pattern. Most importantly, mean landing positions revealed accurate responses for the critical 7.5° targets in all three eccentricity blocks. For most eccentricities, landing positions were narrowly distributed around the target position, with the spread of the distributions increasing with eccentricity (Fig 11, dash-dotted lines).

The mean latency of visually guided saccades was 252 ms. Thus, saccade latencies were longer than in the first two experiments, probably due to the prolonged visual feedback about the target location. The mean latency for memory-guided saccades was 292 ms and hence numerically longer than for visually guided saccades. For each block, a one-sided paired *t* test was run on mean latencies that were obtained by collapsing data across eccentricity conditions. Mean latencies were significantly longer for memory-guided than for visually guided saccades in the Short Block (*t*(11) = 3.8, *p* = .002) and in the Medium Block (*t*(11) = 2.0, *p* = .037). The difference failed to be significant in the Long Block (*t*(11) = 1.5, *p* = .080). Visual inspection of the data presented in Fig 6b suggests that there was no systematic relationship between target eccentricity and saccade latency for memory-guided saccades. For visually guided saccades, there was still a hint of a bowl-shaped latency-eccentricity function, but the pattern was not as clear as in Experiment 2.

## General Discussion

Kapoula [1] proposed that prosaccades exhibit a saccadic range effect such that eye movements towards the proximal and distal targets contained within a stimulus set respectively over- and undershoot the veridical target location. Finding evidence for a SRE would support the view that the range effect generalizes from ballistic arm movements to saccadic eye movements, as proposed by Poulton [3, 4]. This would imply that saccadic eye movements are subject to some general muscle-control bias [3] or strategic adjustments based on the set of conditions in a given experiment [10], rather than being globally adjusted so as to reduce the likelihood of target overshoot [16, 21]. Putting the SRE hypothesis to a test, we failed to find evidence for the effect in three experiments.

### No Evidence for a Saccadic Range Effect

Experiment 1 was a close replication of the original study by Kapoula [1]. Across two additional experiments we manipulated (1) the degree of top-down control evoked by the task and (2) the target presentation duration. If the SRE is a consequence of cognitive control, as proposed by Kapoula, it should be sensitive to top-down requirements associated with the task. Therefore, we expected to replicate the original finding of a SRE for a visual discrimination task [1]. The simple saccade-targeting task in Experiment 3, entailing minimal top-down influence, was expected to elicit a weaker SRE (cf. [23]). Moreover, voluntary saccades to remembered target locations in Experiment 3 were expected to elicit a particularly strong SRE.

Contrary to these predictions, we did not observe a SRE in any of the tasks. Our conclusions were based on analyzing systematic and variable errors in the spatial accuracy of primary saccades. Moreover, the design of Experiment 2 allowed for an additional test of the SRE hypothesis by analyzing properties of secondary saccades. Specifically, we examined the proportion of secondary saccades that corrected for an undershooting or overshooting bias. For the largest eccentricities (≥ 12.5°), secondary saccades were progressively more likely to correct the undershoot response elicited by the primary saccade. However, overshoot correction did not prevail at the nearest eccentricities in a block, which is incompatible with the SRE hypothesis.

Our failures to observe the effect for visually guided saccades are in line with an early study by Findlay [9] and recent reports from another laboratory ([2], see also [14]). Gillen et al. used a task involving minimal top-down control by asking observers to saccade to a cross, which was presented for 50 ms only. Our findings complement theirs by showing that a SRE did not evolve with increasing cognitive demands imposed by the task.

Our three experiments and the experiments by Gillen et al. differed in how long visual information about the target was present after a saccade was launched. Gillen et al. used a very brief (i.e., 50 ms) target presentation. However, Gillen et al. [2] also failed to observe a SRE in a supplemental experiment in which the target was visible until saccade offset. In our experiments, the target remained visible until 100 ms following saccade onset (Exp. 1, as in [1]) or for a fixed period of 500 ms (Exp. 2) or 2 s (Exp. 3). We note that, across experiments, target presentation duration is somewhat confounded with task (Gillen et al. and our Exp. 3: simple saccade-targeting task, Exps. 1 and 2: visual discrimination task). Tian et al. [18] varied how long the target was visible within a single experiment and task. Target presentation duration, ranging from 100 ms to 2 s, did not affect the accuracy of the primary saccade to targets at three different eccentricities. Taking the findings by Tian et al. [18] into account, we tentatively conclude that the presence (or absence) of a saccadic range effect is unlikely to depend on target presentation duration.

Does the SRE depend on the number of trials per block and eccentricity condition? Kapoula [1] reported that “the range effect emerged after a number of trials on a given target set” (p. 1157). In a related study, however, the effect was established rapidly in only a few trials [23]. In Experiment 2, we exposed participants to a greater number of trials than in Experiment 1 (Table 1) to test whether this would strengthen the SRE. In either case, there was no evidence for a SRE. Gillen et al. [2] performed a time-course analysis on their data and showed that the undershooting bias they observed did not change over trials (*N* = 24 per eccentricity condition). We conclude that the null effects reported here cannot be tied to an inadequate number of trials.

A failure to replicate the SRE cannot be attributed to differences in saccade latencies across studies. Mean saccade latencies in Experiments 1 and 2 were similar to mean saccadic reaction times in Kapoula’s [1] study. Furthermore, when saccade latency was inflated as in Experiment 3, there still was no SRE.

In general, a replication failure does not necessarily mean that the original finding is incorrect [28]. However, on closer inspection it becomes apparent that the existing evidence for a SRE is weak at best. Regarding the original study by Kapoula [1], Gillen et al. [2] identified problems in the presentation of her data, concluding that the data do not actually provide evidence for a SRE. In the literature, the study by Kapoula and Robinson [23] is also frequently cited as having provided positive evidence; in addition, there is another study by Kapoula and Bucci [10]. For one, a limiting factor in both studies is that they only tested a single block of target eccentricities, rather than testing different blocks with partially overlapping ranges of target eccentricities. Moreover, there is little evidence for overshoot responses to close targets—a defining feature of the SRE—in these studies. Kapoula and Robinson [23] interpreted their data on the systematic error via descriptive statistics (i.e., means). We conducted inferential analyses of the participant-specific data provided in their Table 1, though we acknowledge that statistical power is low (*N* = 5 subjects). For both the tracking and visual discrimination tasks, there were significant undershoot responses for targets presented at 10, 15, and 20° (all *p* < .02). For the critical 5° eccentricity condition, saccade amplitudes did not significantly differ from veridical target location. Specifically, the small numerical overshoot (mean deviation 0.12°) in the visual discrimination task was not statistically significant, *t*(4) = 0.9, *p* = .411. The small numerical undershoot (mean deviation -0.12°) in the tracking task was also not significant, *t*(4) = -1.1, *p* = .350. In the study by Kapoula and Bucci [10], children with and without strabismus were asked to make a saccade to a target letter at 5, 10, or 15° eccentricity. The children with strabismus were tested before and after surgery under binocular and monocular viewing conditions; the control children were tested in the binocular viewing condition. The article reports that—across viewing conditions—there was no significant difference between saccade amplitude and target location for the middle (10°) eccentricity. The authors report small numerical overshoots for the 5° conditions, but the error bars (standard deviations) overlapped with the veridical target position for the after-surgery and control data. By comparison, the undershoots of targets positioned at 15° were larger in size. Unfortunately, responses to the close (and far) targets were not statistically evaluated. In our opinion, such tests are critical for a test of the SRE hypothesis.

### Replication of Findings from Basic Oculomotor Research

Notably, the data from the present experiments do replicate a number of other phenomena known from basic oculomotor research. The data from Experiments 2 and 3 suggested that the variable error in saccade accuracy, captured by the spread of the landing position distribution, increased as target eccentricity increased (Figs 5 and 11). This finding is consistent with previous research [18, 19, 52]. Analyses of secondary saccades, based on the data from Experiment 2, showed that the probability of a visual target eliciting more than one saccade systematically increased with target eccentricity [20]. In Experiment 2, the latency-eccentricity function had a bowl-shaped form [22, 57]. By comparison, when extensive visual feedback about the target location was available (visually guided saccades in Exp. 3), saccade latencies were prolonged and showed an attenuated latency-eccentricity function.

The results for memory-guided saccades also replicate a number of key findings previously reported in the literature. With regard to the systematic error in saccade programming, our results confirm that saccades to remembered targets are usually more hypometric than visually guided eye movements [17]. Moreover, memory-guided saccades were characterized by a much greater variable error than visually guided saccades [37, 38]. In addition, latencies for memory-guided saccades were longer than for visually guided saccades [17, 37].

### Little Systematic Undershoot

A striking result of the present experiments is that mean landing positions showed little systematic error for visually guided saccades (Figs 3, 4 and 10). In the vast majority of eccentricity conditions, the eyes landed relatively precisely on target, with the proportion of overshoot and undershoot being small and well balanced. Statistical analyses of the data from Experiments 2 and 3 substantiated that only large-eccentricity targets that were presented 15 or 17.5° to the right or left of a previously displayed fixation dot were associated with a systematic undershooting bias. In addition, for the 12.5° eccentricity condition in the Medium Block we observed an undershoot bias in Experiment 2, but not in Experiment 3. When expressed in percent of the distance to the target, the undershoot bias increased with increasing target eccentricity.

In our data, there was no systematic undershooting bias for eccentricities smaller than 12.5°, thereby suggesting that undershooting is not an inevitable property of the saccadic system. A previous study by Frost and Pöppel [20] examined a large range of eccentricities (5–45°) in an “open loop” condition (target presentation 100 ms) and a “closed loop” condition (2 or more seconds). Their data can be summarized as follows. First, only targets beyond 10–15° eccentricity were undershot, and the amount of undershoot increased with target eccentricity. Second, mean saccadic responses for shorter eccentricities were accurate. Third, saccadic accuracy was similar for the two target presentation durations. By and large, the results from the present experiments are compatible with these findings. We note that other studies reported a systematic undershooting bias for smaller eccentricities (3–10.5° in [2]; 10° in [18]; 6–9° in [19]).

### Implications for Eye Movements in Reading

The present results have implications for theories of eye-movement control in reading. In a now classic article, McConkie et al. [51] discovered a linear influence of saccades’ launch-site distance on landing sites for words in sentence reading. If the launch site for a saccade landing on a target word is far from that word, the landing position will be shifted towards the beginning of the word. Similarly, if the launch-site distance is short, the landing position is shifted towards the end of the word. This launch-site effect generalizes to other languages including German [62] and Chinese [63] and is also found when scanning meaningless *z*-strings [64] or symbols in a reading-like sequential search task [65]. The effect appears to be attenuated for large objects embedded in images of real-world scenes [66]. Based on Kapoula’s [1] findings, McConkie et al. adopted the concept of a SRE as an explanation for the launch-site effect in reading. Their propositions—combined with word-based saccade targeting—have been incorporated in several computational models of eye-movement control (SWIFT: [67], E-Z Reader: [68, 69], SERIF: [70], Glenmore: [71]). However, Vitu [72] and Coëffé and O’Regan [56] have explicitly tested the effect of eccentricity on saccade metrics, using isolated words and meaningless letter strings, respectively. They found no evidence for a SRE, though a limiting factor of these studies is that they only tested a single block of target eccentricities. These results and the present findings in particular suggest that authors of computational models should reconsider the SRE explanation for the launch-site effect in sentence reading. Vitu and colleagues suggested an alternative center-of-gravity explanation [55, 73–75]. Another proposal based on McConkie et al.’s assumption of word-based targeting suggests that the launch-site effect in reading is based on Bayesian estimation of saccade target positions [76, 77].

### Saccade Accuracy and Cognitive Strategies

Not finding a range effect in the saccadic system is not to say that cognitive strategies cannot influence the accuracy of saccades. As a matter of fact, the undershooting bias is thought to be a control strategy that minimizes saccade flight time [21] or the energy requirements of the response [16]. Besides, it has been shown that saccadic accuracy and precision can be improved by instructing observers to increase saccade latency and to reach the target with a single saccade [40]. Moreover, visual working memory can influence the metrics of saccades to single targets in peripheral vision [78].

### Conclusion

In summary, the present experiments along with the experiments by Findlay [9] and Gillen et al. [2] failed to find a SRE as reported by Kapoula [1]. We conclude that the SRE for prosaccades towards single peripheral targets is not a robust phenomenon or may not exist after all.

## Supporting Information

S1 Appendix Control Analysis: Saccade Amplitude vs. Landing Position. (DOCX)
