# Supplementary material for: No Evidence for a Saccadic Range Effect for Visually Guided and Memory-Guided Saccades in Simple Saccade-Targeting Tasks
Source: PLoS One. 2016 Sep 22;11(9):e0162449. doi: 10.1371/journal.pone.0162449 (PMC5033472; doi:10.1371/journal.pone.0162449)
Supplement: S1 Appendix — (DOCX) [file pone.0162449.s001.docx]

**S1 Appendix**

Control Analysis: Saccade Amplitude vs. Landing Position

Most previous studies have analyzed saccade amplitude as the dependent measure. In the present article, we analyzed the eyes’ landing position relative to the veridical target position. We reasoned that this may be a more accurate measure as participants’ fixational eye movements during the long start fixation could potentially compromise the validity of saccade amplitude as dependent measure. The aim of the control analysis was to examine the influence of fixational eye movements on the horizontal start position of primary saccades following target presentation. To this end, the mean horizontal deviation of the eyes from the initial fixation dot was computed as a function of block and eccentricity condition, separately for each eye (see Fig A1). Average deviations were in the order of 1 to 2 pixels and thus very small. At the start of the primary saccade towards the target, the right eye consistently fixated somewhat to the right of the fixation dot whereas the left eye fixated slightly to the left of it. Repeated-measures ANOVAs, one for each eye, with block and target eccentricity as factors revealed that these deviations did not systematically vary across block and eccentricity condition [all *F*’s < 1.3, *p* > .05]. These global control analyses suggest that saccade amplitude is as suitable as landing position as dependent measure. Indeed, the pattern of results reported in the main text did not change when saccade amplitude rather than landing position was used as dependent variable.

**Fig A1. Saccade onsets of primary saccades following target presentation.**

The graph shows the horizontal deviation (°) from the fixation dot as a function of eye, block, and target eccentricity condition. The location of the fixation dot is represented by the 0 value on the *y*-axis. Error bars represent within-subject standard errors of the mean.
